# Role of vitellogenins on the mitochondrial unfolded protein response

**DOI:** 10.17912/micropub.biology.002360

**Published:** 2026-09-29

**Authors:** Nathanial A. Jordan, Suzanne Angeli

**Affiliations:** 1 Molecular and Biomedical Sciences, University of Maine

## Abstract

Sex-specific differences influence aging and disease onset. Using *Caenorhabditis elegans* , we previously found that hermaphroditic nematodes activate a robust mitochondrial unfolded protein response (UPR ^mt^ ) in the intestines, while female nematodes do not. We hypothesized that the accumulation of vitellogenins in females was suppressing the UPR ^mt^ . Loss of vitellogenins via RNAi enhanced the UPR ^mt^ in hermaphrodites but had no impact on the UPR ^mt^ of females unless a combination of vitellogenins was simultaneously knocked down. Furthermore, combinatorial loss of vitellogenins did not restore the female UPR ^mt^ to hermaphroditic levels. We conclude that additional factors besides vitellogenins are involved in suppressing the intestinal UPR ^mt^ in female nematodes.

**Figure 1. Impact of vitellogenin RNAi on the UPR mt:** *A-D* . Quantification of GFP intensity from *p hsp-6* ::GFP or *fem-1* ( *hc17* ); p *hsp-6* ::GFP nematodes. For single vitellogenin gene knockdown, nematodes were developed on either control vector (CV) RNAi or *vit-1* ( *A* ), *vit-3* ( *B* ), or *vit-5* ( *C* ) RNAi at 25°C. At the young adult stage, control worms were shifted to 2o°C and onto RNAi plates seeded with CV or CV/ *atp-3* (50%/50% RNAi mixture). Vitellogenin RNAi treated worms were shifted to 2o°C and onto RNAi plates seeded with the indicated CV/ *vit* RNAi or *vit* / *atp-3* (50%/50% RNAi mixture). For the combinatorial vitellogenin gene knockdown ( *D* ), nematodes were developed on either control vector (CV) RNAi or *vit-1* , *vit-3* , *vit-5* (33%/33%/33% mixture) RNAi at 25°C. At the young adult stage, vitellogenin RNAi treated worms were shifted to 2o°C and onto RNAi plates seeded with the CV/ *vit-1 ,3,5* RNAi (50%/50% combinatorial RNAi mixture) or *vit-1 ,3,5* / *atp-3* (50% combinatorial mixture/50% *atp-3* RNAi). Error bars represent ± SEM. *** *p ≤* 0.001, ** *p≤* 0.01, and * *p≤* 0.05.

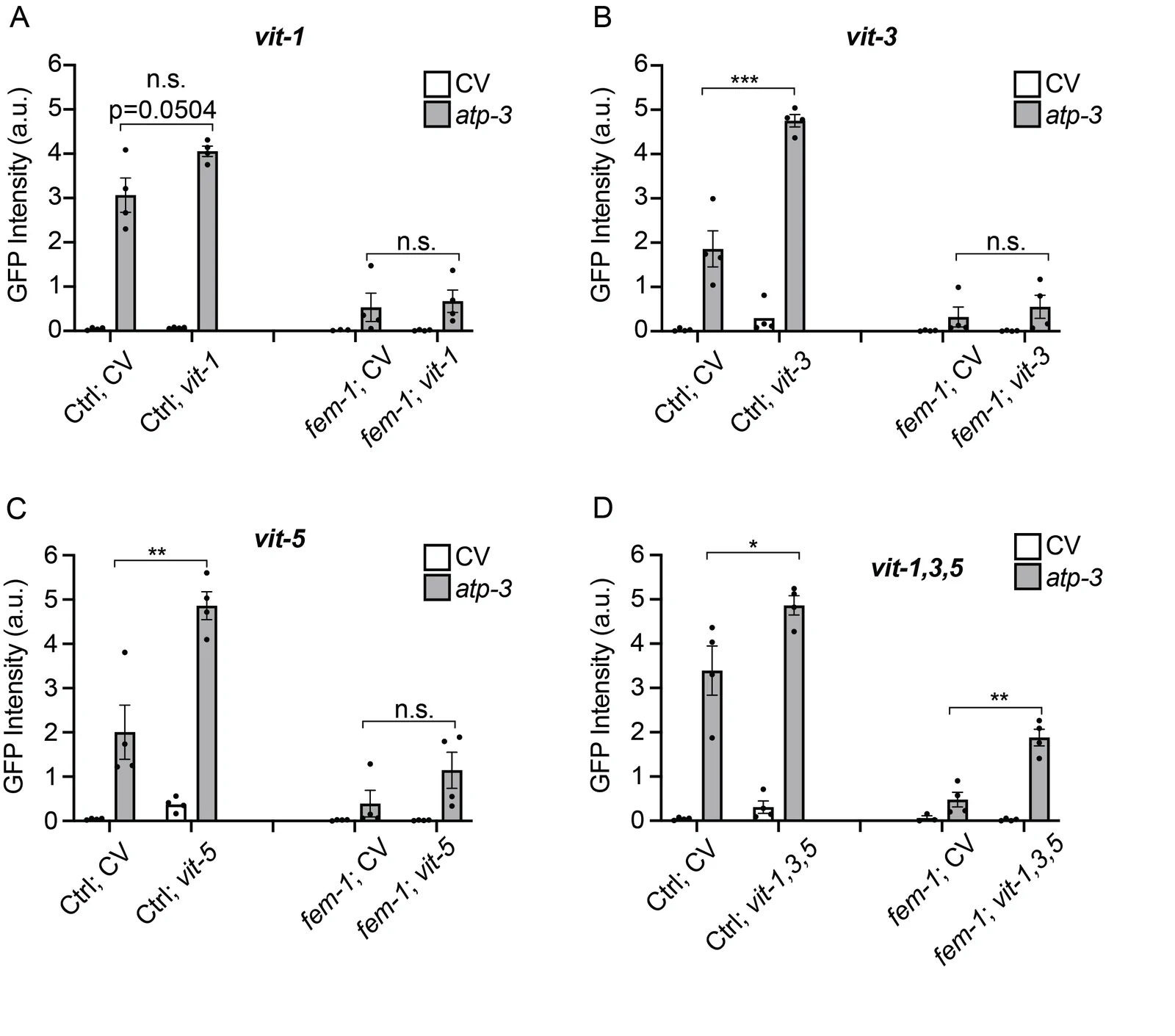



## Description

Age-related diseases, such as cardiovascular disease, cancer, and neurodegenerative syndromes can display sex-specific differences. Since mitochondrial dysfunction is central to most age-related diseases, we examined the mitochondrial unfolded protein response (UPR ^mt^ ), a conserved, broad-range transcriptional response that, among other functions, aids in the refolding of mitochondrial matrix proteins (Kim, Ramalho, and Haynes 2024). In *C. elegans* , UPR ^mt^ activation is largely localized to the intestine, the major metabolic tissue in the nematode. In addition to digestion, the intestine also carries out many functions similar to the liver, such as detoxification, immunity, and fat metabolism (Dimov and Maduro 2019). We and others recently discovered that the activation of the intestinal UPR ^mt^ depends on the function of another tissue: the germline (Foulger et al. 2025; Charmpilas et al. 2024; Shen et al. 2024; Zhou et al. 2024) ^.^ Specifically, we found that adult nematodes with actively proliferating germlines, such as hermaphrodites or mated females, can activate a robust UPR ^mt^ in the intestines when challenged with either a high dose of the metal manganese (Mn) or RNAi of the OSCP/ *atp-3* subunit of complex V (Foulger et al. 2025). Conversely, adult nematodes with mutations that lead to a lack germline stem cells or lack of sperm, such *glp-1* or *fem-1* mutants, cannot activate the intestinal UPR ^mt^ . Additionally, we found that loss of the transcription factor FOXO/ *daf-16* fully restored the UPR ^mt^ in *fem-1* mutants (Foulger et al. 2025). Here, we probe whether vitellogenesis, which occurs in the intestines and is dependent on FOXO/ *daf-16* , impacts the UPR ^mt^ .

Vitellogenins are precursor yolk proteins that provide nutrients to developing embryos. In *C. elegans ,* the highly conserved genes *vit-1* through *vit-6* are responsible for encoding these proteins (Perez and Lehner 2019). In hermaphrodites, they are synthesized in the intestines and transported into the pseudocoelom and to maturing oocytes (Perez and Lehner 2019). Young adult *fem-1* mutants display elevated yolk content compared to wild-type hermaphrodites at Day 1 of adulthood (DePina et al. 2011), presumably due to a lack of vitellogenin transport out of the intestines to maturing oocytes. We used RNAi to knockdown *vit-1* , *vit-3* , *vit-5* , or all three genes simultaneously ( *vit-1 ,3,5* ) and monitored intestinal activation of the UPR ^mt^ in young adult nematodes. We used the UPR ^mt^ reporter in which the GFP expression is driven by the promoter of the mitochondrial chaperone mtHsp70/ *hsp-6* (Yoneda et al. 2004). We additionally used RNAi of OSCP/ *atp-3* to induce the UPR ^mt^ in young adult nematodes as previously described (Angeli et al. 2021). We found that in hermaphroditic nematodes, knockdown of *vit-1* trended toward elevating the intestinal UPR ^mt^ (p=0.0504) and knockdown of *vit-3* , *vit-5* , or *vit-1 ,3,5* all significantly elevated the UPR ^mt^ ( **Figure 1A- 1D** ). In contrast, knockdown of *vit-1* , *vit-3* , or *vit-5* did not significantly alter the UPR ^mt^ of *fem-1* mutants ( **Figure 1A- 1D** ). The knockdown of *vit-1 ,3,5* did significantly enhance the UPR ^mt^ of the *fem-1* mutants, but not to wild-types levels ( **Figure 1D** ). One caveat of this study is that we were not able to procure RNAi clones for *vit-2* , *vit-4* , or *vit-6* . However, since *vit-2* is over 80% identical to *vit-1* and *vit-3* / *vit-4* are duplicated genes that are 99% identical (Perez and Lehner 2019), we would expect to observe more impact from individual RNAi knockdown on females if these vitellogenins played an outsized role on the UPR ^mt^ . The *vit-6* gene is the most divergent vitellogenin, so it remains possible that it plays an untested role in the UPR ^mt^ . Overall, while it does appear that vitellogenins can mildly suppress the adult UPR ^mt^ , especially in hermaphroditic nematodes, we conclude that additional germline-to-intestinal signals are responsible for the potent suppression of the UPR ^mt^ in female nematodes.

## Methods

**Strains.** Bristol N2 (wild type) nematodes were obtained from the *Caenorhabditis* Genetics Center (CGC, University of Minnesota) and cultured using standard conditions (Sulston J 1988). The following strains were used: GL347 ( SJ4100 p *hsp-6* ::GFP backcrossed 6× to N2 ) and GL364 ( *fem-1* ( *hc17* ); p *hsp-6* ::GFP).

**Nematode and bacterial culture conditions.** Nematodes were maintained on nematode growth medium (NGM) plates. NGM plates were seeded with *Escherichia coli* OP50 obtained from CGC that was grown in LB at 37°C for 18 hours shaking at 225 rpm. Seeded plates were dried for 48 hours at room temperature before use. For RNAi experiments, HT115 (DE3) bacteria obtained from the Horizon Biosciences RNAi library were used. All RNAi clones were verified via sequencing. RNAi plates were prepared by cooling NGM to 55°C and supplementing with a final concentration of 50μg/ml carbenicillin and 1 mM isopropyl β-d-1-thiogalactopyranoside (IPTG). RNAi bacteria were inoculated with one colony of RNAi bacteria into LB with 50μg/ml carbenicillin and were grown shaking overnight for 18 hours at 37° at 225 rpm. RNAi cultures were seeded on RNAi plates and allowed to grow for 48 hours at room temperature. Plates were stored at 4 °C for no longer than 2 weeks.

**Microscopy.** Nematodes were anesthetized with 2 - 5mM levamisole and mounted on 2% agarose pads on glass slides. Fluorescence micrographs of GFP were taken using a Zeiss Axioscope 5 fluorescent compound microscope equipped with Zen microscopy imaging program. GFP expression was enhanced using the brightness/contrast tool in Adobe Photoshop. The same parameters were used for all images. GFP intensity of nematodes was quantified using ImageJ 1.54G. The “integrated density” of GFP expression and length of nematodes was measured using ImageJ tools. Integrated density value was normalized by number of nematodes and average length of nematodes. The final value is in arbitrary units.

**Statistics.** Significance between control and experimental groups was determined by using a two-tailed Student's *t* -test. Asterisks denote corresponding statistical significance: * *p <* 0.05; ** *p* < 0.01; *** *p* < 0.001. Error bars were generated using the standard error of the mean (SEM), typically from three or more pooled biological replicates.
